# Major ceRNA regulation and key metabolic signature analysis of intervertebral disc degeneration

**DOI:** 10.1186/s12891-021-04109-8

**Published:** 2021-03-06

**Authors:** Shuai Cao, Jie Li, Kai Yang, Haopeng Li

**Affiliations:** Department of Orthopedics, The Second Affiliated Hospital of Xi’an Jiaotong University, Xi’an, 710004 China

**Keywords:** Intervertebral disc degeneration, Metabolism, Competing endogenous RNA, MicroRNA, Long noncoding RNA

## Abstract

**Background and objective:**

Intervertebral disc degeneration (IDD) is a complex multifactorial and irreversible pathological process. In IDD, multiple competing endogenous RNAs (ceRNA, including mRNA, lncRNA, and pseudogenes) can compete to bind with miRNAs. However, the potential metabolic signatures in nucleus pulposus (NP) cells remain poorly understood. This study investigated key metabolic genes and the ceRNA regulatory mechanisms in the pathogenesis of IDD based on microarray datasets.

**Methods:**

We retrieved and downloaded four independent IDD microarray datasets from the Gene Expression Omnibus. Combining the predicted interactions from online databases (miRcode, miRDB, miRTarBase, and TargetScan), differentially expressed lncRNAs (DElncRNAs), miRNAs (DEmiRNAs), and mRNAs (DEmRNAs) were identified. A ceRNA network was constructed and annotated using GO and KEGG pathway enrichment analyses. Moreover, we searched the online metabolic gene set and used support vector machine (SVM) to find the critical metabolic DEmRNA(s) and other DERNAs. Differential gene expression was validated with a merged dataset.

**Results:**

A total of 45 DEmRNAs, 36 DElncRNAs, and only one DEmiRNA (miR-338-3p) were identified in the IDD microarray datasets. GO and KEGG pathway enrichment analyses revealed that the DEmRNAs were predominantly enriched in the PI3K-Akt signaling pathway, MAPK signaling pathway, IL-17 signaling pathway, apoptosis, and cellular response to oxidative stress. Based on SVM screening, 6-phosphofructo-2-kinase/fructose-2,6-bisphosphatase (*PFK/FBPase*) 2 is the critical metabolic gene with lower expression in IDD, and AC063977.6 is the key lncRNA with lower expression in IDD. The ceRNA hypothesis suggests that AC063977.6, miR-338-3p (high expression), and *PFKFB2* are dysregulated as an axis in IDD.

**Conclusions:**

The results suggest that lncRNA AC063977.6 correlate with *PFKFB2*, the vital metabolic signature gene, via targeting miR-338-3p during IDD pathogenesis. The current study may shed light on unraveling the pathogenesis of IDD.

**Supplementary Information:**

The online version contains supplementary material available at 10.1186/s12891-021-04109-8.

## Background

Intervertebral disc degeneration (IDD), a complex multifactorial and age-dependent condition, is a critical contributor to low-back pain (LBP) [1]. The increasing incidence of IDD not only affects the life quality of patients because of chronic pain and disability, but also causes a severe financial burden to the health care system [2]. Moreover, current prevention, diagnosis, and treatment modalities for IDD are hampered due to its ambiguous pathogenic mechanisms. Previous studies have shown that IDD is characterized by the abnormal proliferation of nucleus pulposus (NP) cells, degradation of proteoglycans and collagen in the extracellular matrix (ECM), imbalance of homeostasis, and accelerated transition of NP cells from a healthy state to a catabolic and degenerative state [3]. It is known that the cellular function of all life forms relies closely on metabolism [4, 5]. Metabolic genes are believed to play essential roles in various cellular functions and many diseases such as hepatic fibrosis, central nervous system diseases, and hematologic malignancies [6–8]. However, investigations concerning the role of metabolic networks in IDD remain sparse. Hence, in-depth comprehension of metabolic gene expression and the regulatory mechanisms of noncoding RNA may provide novel insights into the onset, development, and diagnosis of IDD.

Long noncoding RNAs (lncRNAs), a group of RNA molecules consisting of more than 200 nucleotides with no or feeble protein-coding ability [3], have been of great interest because of their ability to regulate gene expression in various pathological and biological processes, such as cell proliferation and apoptosis [9, 10]. MicroRNAs (miRNAs), consisting of approximately 18–22 nucleotides without coding function, mediate post-transcriptional regulation of target messenger RNA (mRNA) [11]. Ample studies indicate that lncRNAs can act as natural microRNA (miRNA) sponges to bind miRNAs, acting as competing endogenous RNAs (ceRNAs) [12–14]. The ceRNA crosstalk plays a critical role in modulating gene expression [15]. Accumulating evidence has demonstrated the aberrant expression of several mRNAs, miRNAs, and lncRNAs in NP cells in IDD and explained the relationship between them with respect to autophagy, apoptosis, and cell cycle [11, 16, 17]. Nevertheless, metabolic gene-associated ceRNA regulated IDD progression remains largely unstudied. Hence, further exploration of metabolism-related ceRNA crosstalk in NP cells will benefit our understanding of the metabolic gene regulatory network, which is of considerable significance for understanding IDD pathogenic mechanisms. Besides, it is vital to identify the key metabolic gene(s) and relevant key regulated noncoding RNAs in the IDD. In addition, we previously screened core RNAs of IDD based not only on differential expression (DE) but also using the machine learning technology, namely support vector machine (SVM) [18].

Currently, the definitive diagnosis of IDD is sometimes difficult because the intervertebral disc has a particular anatomical structure. In addition, LBP usually has atypical clinical features [19], and the definitive diagnosis is not often achieved using clinical imaging methods [20]. It is even more difficult to correctly diagnose dorsal disc migrations [21], and misdiagnosis often occurs [22]. A transcriptomic signature is beneficial for clear and early diagnosis and timely treatment of patients with IDD [23], especially those that are at a high risk of IDD. Currently, the metabolic gene signature is a collection of metabolism-related genes that can characterize the outcome events; it accounts for the top differentially regulated genes in many disorders but is relatively poorly explored in IDD [24, 25]. In the current study, we aimed to explore the key metabolic gene(s) signature and its key ceRNA regulatory systems to provide insight into IDD pathogenesis. Combining database prediction and gene expression validation, we explored the metabolic abnormalities of NP cells in IDD and revealed their underlying metabolic, molecular signatures and regulation profiles based on microarray datasets.

## Methods

### Data acquisition and study design (Fig. 1)

After searching the National Center of Biotechnology Information (NCBI) Gene Expression Omnibus (GEO) database (https://www.ncbi.nlm.nih.gov/geo), we retrieved and downloaded all four IDD microarray datasets (lncRNA microarray set, GSE56081; miRNA microarray sets GSE116726 and GSE19943; and mRNA microarray sets, GSE56081 and GSE70362). According to the Thompson grading system, grade I to grade III NP samples were classified as control samples, and grade IV and grade V NP samples were classified as IDD samples [26]. GSE56081 consists of five IDD and five control samples, whereas GSE116726 and GSE19943 include three IDD and three control samples, respectively. The GSE70362 consists of 14 control samples and 10 IDD samples (Table 1). In order to obtain standardized data, we performed batch normalization on GSE116726 and GSE19943 using the R package ‘sva’. For mRNA analysis, the GSE56081 microarray set was used for initial exploration, and the GSE70362 microarray set was normalized, merged and used for further validation (batch normalization and merging with GSE56081 to obtain large sample size). Because the data were publicly obtained from the GEO database, we strictly followed the publication guidelines approved by GEO (details with platform information, sample size, and access are shown in Supplementary Table 1. The characteristics of IDD patients in related GEO datasets are shown in Supplementary Table 2). And, ethics committee approval was not required to conduct this study.

**Fig. 1:**
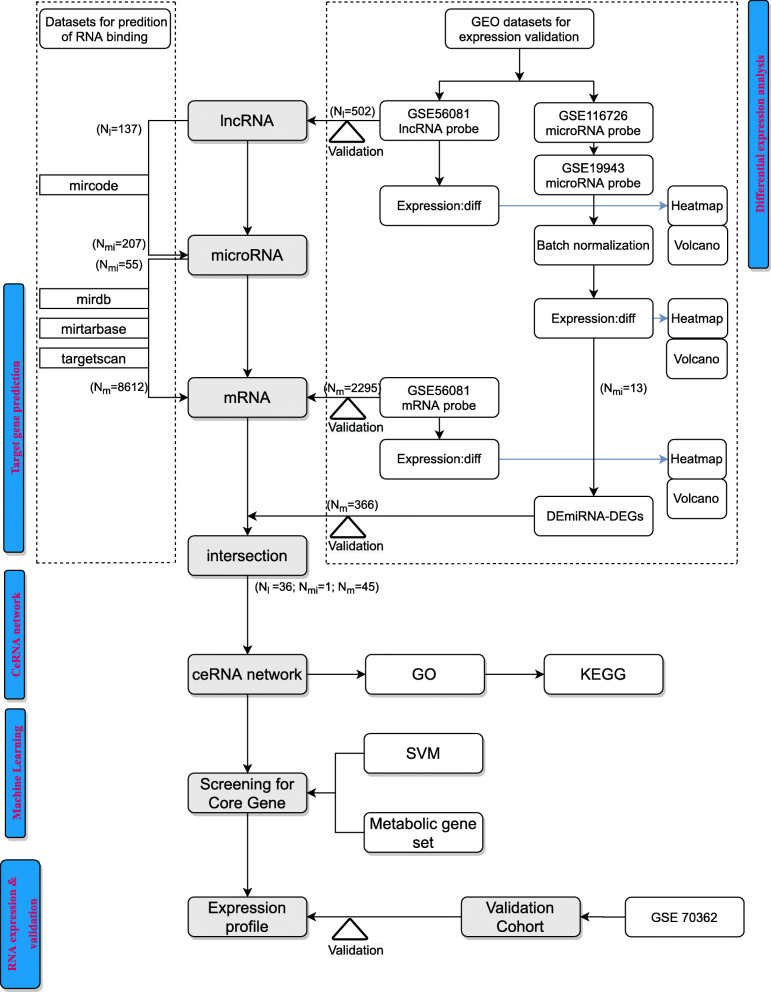
Flowchart of our bioinformatic analysis. The logical steps: 1. Differential expression analysis; 2. Target gene prediction; 3. ceRNA network construction; 4. Machine learning (for screening core gene); 5. RNA expression and validation. Intersection means the set of common gene IDs in different gene sets

**Table 1:** Details and sample size with studied GEO datasets

Datasets	Platform information	Control(N)	IDD(N)	Application
GSE56081	GPL15314 Arraystar Human LncRNA microarray V2.0	5	5	identification of DElncRNA
GSE116726	GPL20712 Agilent-070156 Human miRNA [miRNA version]	3	3	identification of DEmiRNA
GSE19943	GPL9946 Exiqon human miRCURY LNA™ miRNA Array V11.0	3	3	identification of DEmiRNA
GSE56081	GPL15314 Arraystar Human LncRNA microarray V2.0	5	5	identification of metabolic DEGs
GSE70362	GPL17810[HG-U133_Plus_2] Affymetrix Human Genome U133	14	10	validation of metabolic DEGs

*Abbreviations*: *GEO* Gene Expression Omnibus, *IDD* Intervertebral disc degeneration, *DElncRNAs* differentially expressed lncRNAs, *DEmiRNAs* differentially expressed miRNAs, *DEGs* differentially expressed genes

### DE lncRNAs, miRNAs, mRNA screening

The ceRNA network was constructed based on DE analysis for differentially expressed lncRNAs (DElncRNAs), differentially expressed miRNAs (DEmiRNAs) and differentially expressed mRNAs [DEmRNAs, responding to differentially expressed genes (DEGs)] of microarrays with R package ‘limma’ and the prediction for interactions in the online database. First, DElncRNAs were identified between IDD and control samples in GSE56081. Next, a highly conservative miRNA family profile on the Mircode database (http://www.mircode.org/) was used to predict interactions between miRNAs for the above DElncRNAs. To validate and ensure the reliability and accuracy of the predicted data, we obtained another set of DEmiRNAs in IDD samples from GSE116726 and GSE19943 for comparison, with the thresholds of *P* < 0.05 (two-tailed) and |log_2_ fold change (FC)| ≥ 1. Then, the overlapped miRNAs were used for the final DElncRNA screening.

### Prediction of DEmRNAs

**Fig. 2:**
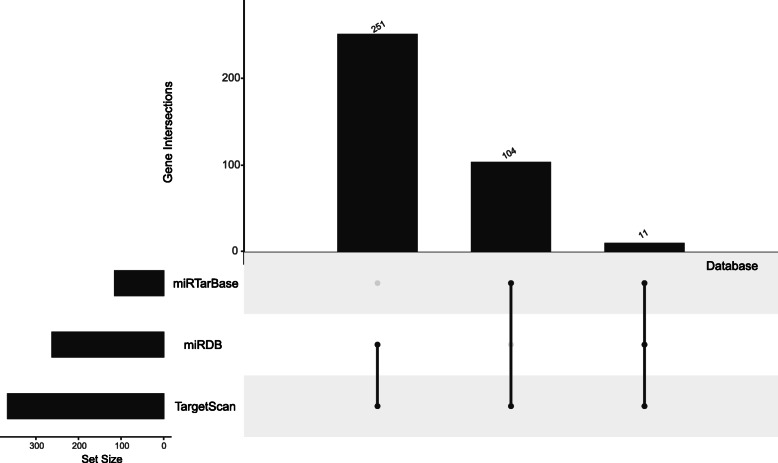
The method for genes scan of miRNAs. Targets of DEmiRNAs were predicted by three experimentally independent databases, including miRDB (http://mirdb.org/), miRTarBase (http://mirtarbase.mbc.nctu.edu.tw/php/index.php) and TargetScan (http://www.targetscan.org/vert_72/). The target gene is the one at least overlapping by two of three binding prediction databases. Intersection means that the number of overlapping genes in different databases (2 or 3)

As shown in Fig. 2, DEmiRNA targets were predicted using three independent experimental databases, including miRDB (http://mirdb.org/), miRTarBase (http://mirtarbase.mbc.nctu.edu.tw/php/index.php), and TargetScan (http://www.targetscan.org/vert_72/). Here, a scoring system was used to predict genes in the DEmRNAs group (DEmRNAs-1). One score was added to the target (mRNA) for each above database that could interact with each other. If the score was ≥2, the mRNA was put into the DEmRNAs-1 group. Meanwhile, the DEmRNAs-2 group was directly identified in GSE56081. Besides, the targets of the overlapped miRNAs from datasets GSE116726 and GSE19943 were predicted as the DEmRNAs-3 group. Next, we kept the overlapped mRNAs in all DEmRNA groups (DEmRNAs-1, DEmRNAs-2, and DEmRNAs-3) as the final DEmRNAs in this study.

After the DE analysis process (Fig. 1), we visualized DElncRNAs, DEmiRNAs, and DEGs between IDD and control samples using clustering heat maps and volcano maps, respectively, using the R package ‘pheatmap’. The key DElncRNAs, DEmiRNAs, and DEGs were selected as the sources of the ceRNA network, which was constructed using Cytoscape3.8.0 [27]. Dark blue rectangle nodes represent the lncRNAs in the network, the purple rhombus nodes represent the target genes, and lines indicate interactions.

### Functional enrichment analyses of the ceRNA network

We performed Gene Ontology (GO) and Kyoto Encyclopedia of Genes and Genomes (KEGG) pathway enrichment analyses of DEGs using R packages (“clusterProfiler”, “org. Hs.eg.db”, “enrichplot”, and “ggplot2” package) with identified filters of *p-*Value < 0.05 and q-Value < 0.05 [28–30].

### Feature selection based on SVM and metabolic gene set

Support vector machine (SVM), a machine learning technology, is a supervised learning model with associated learning algorithms that analyzes data used for classification and regression analyses. Based on the expression levels of the ceRNA, we used the R package ‘caret’ and adopted 5-fold cross-validation to conduct the SVM model for screening vital RNAs. The random seed was set to 39 in all SVM progress. Moreover, we searched for genes on the metabolic gene set based on metabolism-related pathways, which were downloaded from the gene set enrichment analysis (GSEA) website (GSEA, c2.cp.kegg.v7.1.symbols.gmt; https://www.gsea-msigdb.org/gsea/downloads.jsp). We obtained 948 metabolism-related genes for follow-up analysis (Supplementary Table 3). Finally, we used the Venn method (http://bioinformatics.psb.ugent.be/webtools/Venn/) to search for the key DEG(s) from the results of SVM, expression levels, and the metabolic gene set. Furthermore, we searched for other differentially expressed RNAs (DERNAs) based on the SVM and expression levels to find the critical ceRNAs regulated by the key metabolic gene(s).

### Statistical analyses

The R software (version 3.6.2, The R Foundation for Statistical Computing, Vienna, Austria) and MedCalc statistical software (Version 19.0.4, MedCalc Software bvba, Ostend, Belgium) were used for the statistical analyses. Prism (version 8.0, GraphPad Prism Inc.) and R software were used for graphics. DElncRNAs, DEmiRNAs, and DEmRNAs between IDD and control samples were identified using filters with *P* < 0.05 (two-tailed) and |log_2_FC| ≥ 1. A positive log_2_FC value indicated upregulated expression, whereas a negative log_2_FC value indicated downregulated expression. The chi-square test was used for enrichment analyses. Difference with *P* < 0.05 were considered statistically significant.

## Results

### Identification of DElncRNAs, DEmiRNAs, and DEGs using integrated microarray analysis

**Fig. 3:**
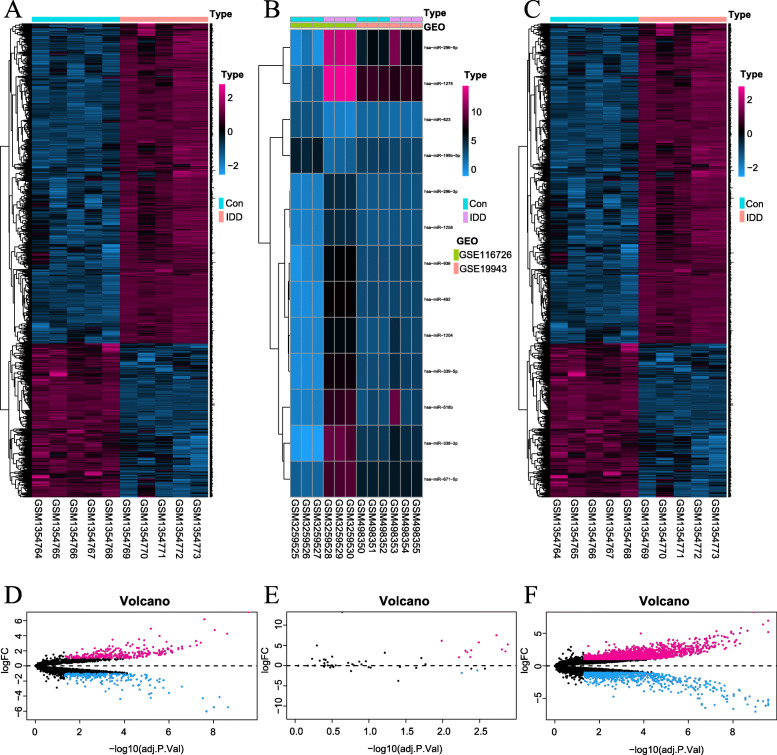
Difference analysis. **a**-**c** The differentially expressed heat-maps of lncRNA, microRNA, and mRNA. The lncRNA heat-map of GSE56081 dataset (**a**); The merged microRNA heat-maps of GSE116726 and GSE19943 datasets (**b**); The mRNA heat-map of GSE56081 dataset. **d**-**f** The volcano plot (**c**). The lncRNA dataset of GSE56081 (**d**); The merged microRNA datasets of GSE116726 and GSE19943 (**e**); The mRNA dataset of GSE56081 (**f**). The screening condition: absolute values of log (fold change) > 1.0 and adjusted *p*-Value < 0.05. In the plots, the rose red and blue dots represent high and low RNA expressions with statistical significance between the IDD NPs and the normal NPs, respectively. And, the black dots represent no statistical significance between them

A total of 502 DElncRNAs and 2295 DEmRNAs were directly identified on GSE56081. Moreover, 15 DEmiRNAs were identified in GSE116726 and GSE19943 (Figs. 1 and 3).

### Target prediction for DElncRNAs, DEmiRNA, and DEGs

Of the 502 DElncRNAs, 137 could bind to 207 miRNAs. Target prediction for the above 207 miRNAs revealed that 55 miRNAs could predict the downstream target mRNAs (DEmRNAs-1 group). Surprisingly, we found that miR-338-3p was the only overlapping miRNA between the 55 miRNAs and the 15 DEmiRNAs. Thus, focusing on miR-338-3p, we mined 36 upstream lncRNAs that could bind to it. Furthermore, 366 downstream mRNAs (DEmRNAs-3 group) related to miR-338-3p were also predicted. There were 8612 target mRNAs (DEmRNAs-1 group) of the 55 miRNAs, which were predicted using the miRDB, miRTarBase, and TargetScan databases (Fig. 2). Finally, 45 DEmRNAs (final DEmRNAs, Supplementary Table 4), which bind to miR-338-3p, were the overlapping genes among the 366 mRNAs, 8612 mRNAs, and 2295mRNAs. The 45 DEmRNAs, which combine with miR-338-3p, were concentrated.

**Fig. 4:**
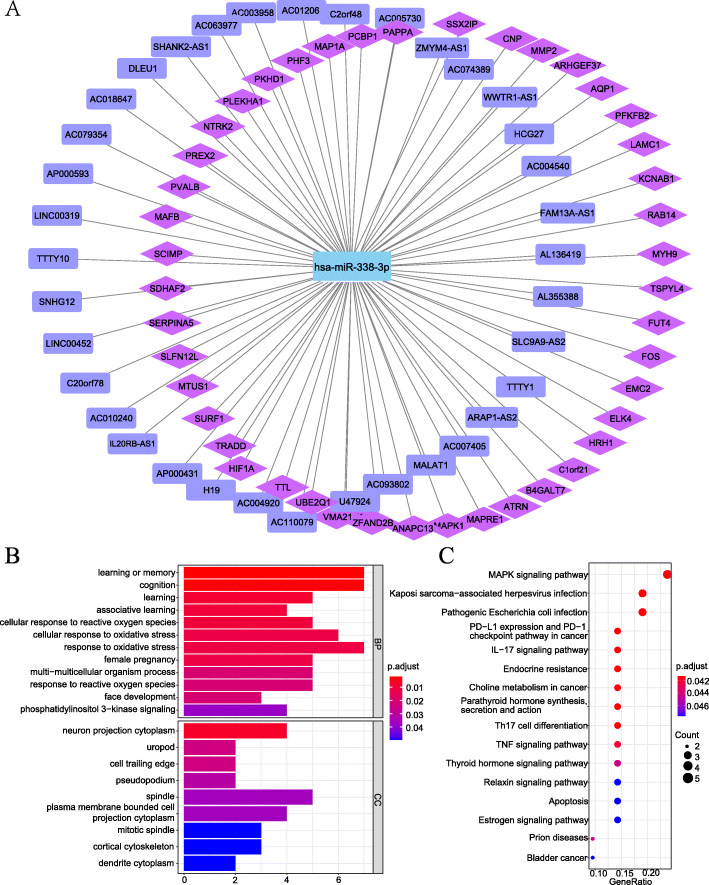
The ceRNA network and its enrichment analysis by GO annotation and pathways identified by KEGG for Differentially expressed genes. The ceRNA network created by Cytoscape 3.8.3 (**a**). The connections DElncRNA (purple rectangle), DEmicroRNA (blue), and target genes (purple pink rhombus) were represented as nodes, and their interactions were denoted by lines. The top terms of enriched GO analysis (**b**). The top terms of enriched KEGG pathway [28–30] (**c**). Abbreviation: GO, the gene ontology; KEGG, the Kyoto encyclopedia of genes and genomes; BP, the biological process; CC, the cellular component. The details for actual gene IDs and GO descriptions were shown in Supplementary Table 5. The details for actual gene IDs per KEGG pathway were shown in Supplementary Table 6. We are grateful to Kanehisa Laboratories

To reveal the underlying molecular signatures of IDD, we constructed a ceRNA network using overlapped DElncRNAs and DEGs, the core of which was miR-338-3p. The ceRNA network displayed 45 miR-338-3p/lncRNA links and 36 miR-338-3p/mRNA links (Fig. 4a).

### Functional enrichment analyses

The GO terms (45 DEGs) indicated that several genes, which existed in the cell trailing edge, were enriched in the phosphatidylinositol 3-kinase signaling pathway and the cellular response to reactive oxidative stress in biological process (Fig. 4b, details of actual gene IDs and GO descriptions are shown in Supplementary Table 5). Moreover, the KEGG pathway enrichment analysis showed that the 45 DEGs were predominantly enriched in 16 pathways, including the MAPK signaling pathway, IL-17 signaling pathway, apoptosis, and TNF signaling pathway, et al. (Fig. 4c) The details of actual gene IDs per pathway are shown in Supplementary Table 6.

### Feature selection based on SVM and metabolic gene set

**Fig. 5:**
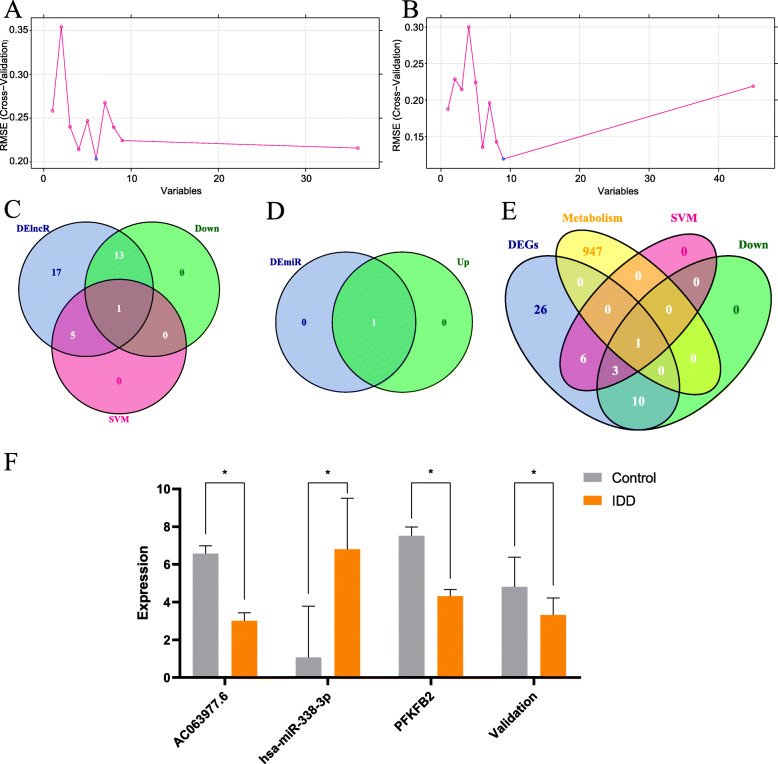
Screening for key metabolic gene and associated ceRNAs. SVM for screening core DElncRNA (**a**). SVM for screening core DEGs (**b**). A 3 set Venn diagram (**c**). The pink circle represents for results of SVM with DElncRNAs, the blue circle represents for DElncRNAs, and the green circle represents for genes with low expression. A 2 set Venn diagram (**d**): the blue circle represents for batch normalized DEmiRNAs, and the green circle represents for genes with high expression. A 3 set Venn diagram (**e**): the pink circle represents for results of SVM with DEGs, the blue circle represents for DEGs, the yellow circle represents for dataset of metabolic genes, and the green circle represents for genes with low expression. The expression profile of metabolic gene and associated ceRNAs in cotrol cohort and IDD cohort (AC063977.6, miR-338-3p, PFKFB2,). Expressional values are means ± SD; *, *p* < 0.05 (**f**). The expression profile also numerically shown in supplementary Table 7. Validation represents the *PFKFB2* expression profile in validation dataset (GSE70362 merging GSE56081)

In dataset GSE56081, we defined 45 overlapping DEGs. Moreover, the metabolic gene set consisted of 948 genes. Hence, we used the SVM method to build a classifier and create a decision boundary for the 36 DElncRNAs and 45 DEGs (Fig. 5a, b). SVM was not used for screening DEmiRNAs because miR-338-3p was the only core DEmiRNA. Then, the Venn method was used to search for the key DElncRNAs and DEGs from the results of SVM signature, expression levels, and the metabolic gene set (Fig. 5c, d, e). The core DElncRNA was lncRNA AC063977.6 (Fig. 5c). The core metabolic gene was 6-phosphofructo-2-kinase/fructose-2,6-bisphosphatase 2 (*PFK/FBPase2*) after training by SVM and intersecting with the metabolic gene set (Fig. 5e). The expression levels of core RNAs are presented as a histogram (Fig. 5f).

## Discussion

The metabolic homeostasis and the construction of the ECM of intervertebral discs mainly depend on its active NP cells, which form the inner core of the intervertebral disc. An imbalance of cellular functions because of metabolic imbalance, nutrient deprivation, dysregulated apoptosis, and some transcellular signaling interrupts homeostasis in NP cells, resulting in IDD [31–33]. Furthermore, the metabolic signature represents a broad molecular signature of systemic metabolism and covers multiple cellular metabolic pathways [34]. The metabolic signature and its relevant mechanism are critical for understanding the cellular mechanisms of various diseases. Accordingly, it would be reasonable to assume that NP cell metabolic disorder might be a pathogenic factor of IDD. However, the metabolic investigations and associated ceRNA regulation mechanism of NP cells in IDD remaine inconclusive. The ceRNAs regulate the expression of various genes and play a significant role in the pathogenesis of many diseases [35]. Identifying ceRNA regulation with the metabolic signatures in NP cells would shed a novel light on IDD. Thus, based on the GEO dataset, we identified a novel gene metabolic signature and related ceRNA regulatory mechanisms to gain insight into the IDD pathogenic mechanism.

In the present study, 45 DEGs and 36 DElncRNAs were identified. MiR-338-3p was the only key DEmiRNA, which was upregulated in IDD. A vast ceRNA network was constructed to reveal the underlying molecular signatures of IDD. GO annotation revealed that the DEmRNAs/DEGs were mostly enriched in the PI3K-Akt signaling pathway. The PI3K-Akt signaling pathway participates in the synthesis of ECM, regulating apoptosis and cell proliferation in IDD. Furthermore, the GO annotation showed that many biological processes were categorized in response to oxidative stress of NP cells, which indicates that oxygen and oxygen-associated processes might play an essential role in IDD. The KEGG pathway enrichment analysis showed that the MAPK signaling pathway, IL-17 signaling pathway, apoptosis, and TNF signaling pathway might be related to IDD. Based on batch normalization, we found that miR-338-3p was the only DEmiRNA validated in two datasets and was correctly predicted for lncRNA-miRNA and mRNA-miRNA binding. However, there were 45 overlapping DEGs and 948 genes defined by the metabolic gene set. To further determine the key metabolic signature and critical lncRNA(s) of the 36 DElncRNAs in IDD, we used the SVM method. Thus, AC063977.6 was identified as the critical lncRNA with recursive feature elimination, based on the results of SVM and related expression regulation of DElncRNAs. Moreover, *PFKFB2* is the key metabolic gene with recursive feature elimination, based on the results of SVM, metabolic gene set, and related expression regulation of DEmRNAs.

According to the online database prediction, AC063977.6 directly targets miR-338-3p, whereas miR-338-3p directly targets *PFKFB2*, suggesting that AC063977.6, miR-338-3p, and *PFKFB2* might form an axis that modulates NP cell metabolism in IDD. Furthermore, the gene expression validation based on microarray data of GSE116726 and GSE19943 further enhanced the credibility of this axis. In conclusion, we found that AC063977.6, miR-338-3p, and *PFKFB2* formed an axis in IDD to modulate the metabolism of NP cells. This study revealed that miR-338-3p was upregulated in degenerating NP cells, accompanied by downregulation of *PFKFB2* expression. Moreover, AC063977.6, acting as a ceRNA sponging miR-338-3p, was significantly attenuated in IDD progression.

The central part of the DEGs is *PFKFB2*, which is an isoform of the PFKFB family that mainly regulates glycolytic metabolism and catalyzes the fructose 2,6-bisphosphate (Fru-2,6-P) [36]. PFKFB2 is a key bifunctional enzyme involved in glycolytic metabolism [37]. Moreover, glycolysis significantly diminishes cellular oxidative stress [38, 39]. Similarly, bioinformatics analysis with the GEO microarray datasets revealed that the DEGs were significantly enriched in the oxidative stress process in GO enrichment. Compared to normal cells, several cancer cells exhibit elevated expression levels of *PFKFB* [40]. However, in this study, we found that the expression level of *PFKFB2* in NP cells with IDD was lower than that in control NP cells, suggesting that reduced glycolysis may increase the level of cellular oxidative stress in NP cells; thus, it may be associated with IDD.

Accumulating evidence suggests that lncRNAs and miRNAs dysregulate essential pathological processes of IDD, such as apoptosis, angiogenesis, ECM degradation, and inflammatory responses [41]. The miR-338-3p originates from the apoptosis-associated tyrosine kinase gene, and it is dysregulated in many tumors and plays distinct roles in different diseases [42]. The upregulation of miR-338-3p could promote glioma cell invasion and metastasis of lung cancer, whereas the downregulation of miR-338-3p was related to poor outcomes in gastric cancer [43–45]. In this study, miR-338-3p was remarkably upregulated in IDD, serving as an impetus with metabolic dysregulation by targeting *PFKFB2*, thereby disturbing the metabolism of NP cells. IDD severely threatens the health of patients, and identification of genetic signature is beneficial for its early diagnosis and early treatment [23]. Moreover, a strong negative correlation was observed between IDD and *PFKFB2* expression levels in both the training cohort and validation cohort, which indicates that low expression of *PFKFB2* is a key factor in IDD. The results suggest that *PFKFB2* is an player in IDD. According to our experience in clinical work and reported literature, misdiagnosis of IDD occasionally occurs, which often leads to incorrect treatment and even unnecessary surgery. Based on our results, the metabolic signature could be used in providing clues for IDD mechanism research, and may aid in surgery for differential diagnosis in clinical practice.

### Limitations of this study

There are some limitations to this study. First, we focused only on the regulatory roles of target genes without further classification into specific subgroups according to their functions, which limited the excavation of those data. Second, further in vitro and in vivo experiments on the sophisticated regulating mechanism involved with AC063977.6 and miR-338 in IDD progression are urgently needed. Third, although a small sample size is a common problem in orthopedic research, it is undeniable that this study is based on a small sample size (a total of 46 samples were included). Sufficient in vitro and in vivo experiments are warranted to establish the functions and mechanisms of the lncRNAs and miRNAs involved in the pathogenesis of IDD and may further promote the translation of precise gene diagnosis in IDD.

## Conclusion

The results suggest that lncRNA AC063977.6, acting as a ceRNA that sponges miR-338, correlating with the metabolism of NP cells via the AC063977.6/miR-338/*PFKFB2* axis in IDD. *PFKFB2* can serve as a metabolic biomarker to contribute to the differential diagnosis of IDD in clinical practice.

## Supplementary Information

**Additional file 1: Table S1.** Details with platform information, sample size, and access in studied GEO datasets. **Table S2.** The characteristics of IDD patients in related datasets. **Table S3.** The 948 metabolism-related genes/ The metabolic gene set. **Table S4.** The final 45 DEmRNAs (or DEGs). **Table S5.** The details for actual gene IDs and GO descriptions. **Table S6.** The details for actual gene IDs per pathway. **Table S7.** Expression profile.

## Data Availability

The datasets generated during the current study are available in the GEO repository [https://www.ncbi.nlm.nih.gov/geo]. The datasets used and/or analyzed during the current study are also available from the corresponding author upon reasonable request. The authors declare that the databases described in the manuscript are available for testing. Details pertaining to the dataset platform information, sample size, and access are shown in Supplementary Table 1.

## Abbreviations

IDD: Intervertebral disc degeneration
miRNAs: MicroRNAs
lncRNAs: Long noncoding RNAs
ceRNA: Competing endogenous RNA
NP: Nucleus pulposus
GEO: Gene Expression Omnibus
DE: Differential expression
DElncRNAs: Differentially expressed lncRNAs
DEmiRNAs: Differentially expressed miRNAs
DEmRNAs: Differentially expressed mRNAs
SVM: Support vector machine
PFKFB: 6-phosphofructo-2-kinase/fructose-2,6-bisphosphatas
AUC: The area under curve
LBP: Low-back pain
ECM: Extracellular matrix
mRNA: Messenger RNA
DEGs: Differentially expressed genes
GO: Gene Ontology
KEGG: Kyoto Encyclopedia of Genes and Genomes
ROC: Receiver operating characteristic
