# Supplementary material for: Major ceRNA regulation and key metabolic signature analysis of intervertebral disc degeneration
Source: BMC Musculoskelet Disord. 2021 Mar 6;22:249. doi: 10.1186/s12891-021-04109-8 (PMC7937257; doi:10.1186/s12891-021-04109-8)
Supplement: Supplementary file 1 — Additional file 1: Table S1. Details with platform information, sample size, and access in studied GEO datasets. Table S2. The characteristics of IDD patients in related datasets. Table S3. The 948 metabolism-related genes/ The metabolic gene set. Table S4. The final 45 DEmRNAs (or DEGs). Table S5. The details for actual gene IDs and GO descriptions. Table S6. The details for actual gene IDs per pathway. Table S7. Expression profile. [file 12891_2021_4109_MOESM1_ESM.docx]

**Major ceRNA regulation and key metabolic signature analysis of intervertebral disc degeneration**

Shuai Cao^1^, Jie Li^1^, Kai Yang^1^, Haopeng Li^✉^

**Supplementary Table 1.** Details with platform information, sample size, and access et al. in studied GEO datasets.

| **Datasets** | **Platform** | **Control** | **IDD** | **Contact name** | **Address** | **Submission** | **Application** | **Access** |
| --- | --- | --- | --- | --- | --- | --- | --- | --- |
| GSE56081 | GPL15314 | 5 | 5 | Hai-Qiang Wang[1-3] | Shaanxi | 2014 | Identification for DElncRNA; Identification for metabolic DEGs | https://www.ncbi.nlm.nih.gov/geo/query/acc.cgi?acc=GSE56081 |
| GSE116726 | GPL20712 | 3 | 3 | Jian Chen[4] | Shanghai | 2018 | Identification for DEmiRNA | https://www.ncbi.nlm.nih.gov/geo/query/acc.cgi?acc=GSE116726 |
| GSE19943 | GPL9946 | 3 | 3 | Hai-Qiang Wang[5] | Shaanxi | 2010 | Identification for DEmiRNA | https://www.ncbi.nlm.nih.gov/geo/query/acc.cgi?acc=GSE19943 |
| GSE70362 | GPL17810 | 14 | 10 | Peadar O'Gaora[6] | Dublin | 2015 | Validation for metabolic DEGs | https://www.ncbi.nlm.nih.gov/geo/query/acc.cgi?acc=GSE70362 |

Abbreviations: IDD, Intervertebral disc degeneration; DElncRNAs, differentially expressed lncRNAs; DEmiRNAs, differentially expressed miRNAs; DEGs, differentially expressed genes; GEO: Gene Expression Omnibus (<https://www.ncbi.nlm.nih.gov/geo>).

**References:**

1. Lan, P.H., et al., Landscape of RNAs in human lumbar disc degeneration. Oncotarget, 2016. 7(39): p. 63166-63176.

2. Liu, X., et al., Noncoding RNAs in human intervertebral disc degeneration: An integrated microarray study. Genom Data, 2015. 5: p. 80-1.

3. Wan, Z.Y., et al., Aberrantly expressed long noncoding RNAs in human intervertebral disc degeneration: a microarray related study. Arthritis Res Ther, 2014. 16(5): p. 465.

4. Ji, M.L., et al., Preclinical development of a microRNA-based therapy for intervertebral disc degeneration. Nat Commun, 2018. 9(1): p. 5051.

5. Wang, H.Q., et al., Deregulated miR-155 promotes Fas-mediated apoptosis in human intervertebral disc degeneration by targeting FADD and caspase-3. J Pathol, 2011. 225(2): p. 232-42.

6. Kazezian, Z., et al., Gene Expression Profiling Identifies Interferon Signalling Molecules and IGFBP3 in Human Degenerative Annulus Fibrosus. Sci Rep, 2015. 5: p. 15662.

**Supplementary Table 2.** The characteristics of IDD patients in related datasets.

| Datasets | Cohorts | Samples | Female | Male | Age(average) |
| --- | --- | --- | --- | --- | --- |
| GSE56081 |  |  |  |  |  |
|  | Control | 5 | 1 | 4 | 40.8 |
|  | IDD | 5 | 2 | 3 | 36.8 |
| GSE116726 |  |  |  |  |  |
|  | Control | 3 | 0 | 3 | 56 |
|  | IDD | 3 | 0 | 3 | 56 |
| GSE19943 |  |  |  |  |  |
|  | Control | 3 | 3 | 0 | 19.3 |
|  | IDD | 3 | 3 | 0 | 30.7 |
| GSE70362 |  |  |  |  |  |
|  | Control | 14 | 7 | 7 | 48.9 |
|  | IDD | 10 | 3 | 7 | 74.8 |

**Supplementary Table 3.** The 948 metabolism-related genes/ The metabolic gene set.

| MPI | PGM1 | ADH5 | DGUOK | PAICS | POLR3D | AK3 | SARDH | NAGS | IDO2 | GSTM4 | HEXB | CEL | PI4KB | CHKB | SGPP2 | DLAT | BST1 | COX15 |
| --- | --- | --- | --- | --- | --- | --- | --- | --- | --- | --- | --- | --- | --- | --- | --- | --- | --- | --- |
| PMM2 | B4GALT2 | ACSL5 | POLR3C | NT5C1A | RRM2 | GLUD2 | AMT | P4HA3 | AANAT | GGCT | HEXA | GPAT2 | PIK3CG | PISD | GALC | MDH1 | NNT | HMBS |
| PMM1 | GAA | ADH1A | NME3 | POLR2B | NT5C3A | GFPT2 | CHDH | OTC | WARS | GSTM3 | GNE | LIPF | INPP5E | PLD2 | SGMS2 | PC | CD38 | CP |
| FBP2 | UGT1A10 | EHHADH | POLR3G | POLR3A | PDE8B | AGXT2 | CBS | PRODH2 | IDO1 | GSTM2 | NAGK | PLCB2 | OCRL | PCYT1B | GBA | GLO1 | NADSYN1 | EARS2 |
| PFKM | UGT1A8 | GCDH | POLR3F | AC020613.1 | POLR3GL | CPS1 | CTH | OAT | CAT | GSTM5 | CHIT1 | PLCB1 | PI4KA | LCAT | SPHK2 | ACACB | NUDT12 | EPRS |
| GMDS | UGT1A7 | ACOX3 | PRUNE1 | POLR2A | NT5E | GLS2 | PIPOX | CKM | OGDH | GSTA1 | UAP1 | INPP1 | PEMT | CRLS1 | NEU2 | ACACA | NMRK1 | CA5A |
| PFKFB4 | UGT1A6 | ACSL1 | ADPRM | AC139530.1 | NPR2 | ABAT | DAO | ACY1 | TPH1 | GSTA2 | RENBP | IPMK | PLA2G15 | GPD2 | NEU1 | ACOT12 | QPRT | CA5B |
| PFKL | ALDH1B1 | ACAA1 | PDE4B | POLR2D | NPR1 | GLUD1 | AMD1 | SAT2 | AFMID | GSR | CHIA | PLCD1 | GPD1 | ENPP2 | ACER2 | ACO1 | NAMPT | CA6 |
| MTMR6 | UGT2B28 | CPT2 | AC005759.1 | DCK | PDE10A | GLS | SRM | CKMT1B | CYP1A1 | GSS | NPL | PLCB3 | GPD1L | PAFAH1B1 | UGCG | MTHFD2 | NMNAT3 | CA12 |
| TPI1 | ALDH2 | CPT1B | PDE3B | POLR2C | ENTPD6 | ASL | ADI1 | PYCR3 | CYP1A2 | GCLC | CMAS | PLCB4 | LPGAT1 | ENPP6 | DEGS2 | CS | NMNAT2 | CA7 |
| PHPT1 | UGT1A5 | ACOX1 | PDE4A | ADCY8 | ENTPD2 | IL4I1 | AHCY | PRODH | CYP1B1 | GSTK1 | PGM3 | INPP5A | MBOAT7 | AGPS | ARSA | HAO1 | NADK | CA2 |
| PFKFB3 | MIOX | ECI2 | NUDT2 | ADCY9 | ENTPD5 | GPT | DNMT1 | CKMT2 | ASMT | GPX5 | GNPDA1 | INPP5B | CHAT | PAFAH1B3 | SPHK1 | MTHFD2L | NNMT | CA3 |
| FCSK | UGDH | ECI1 | GMPR2 | ADCY6 | ENTPD3 | GFPT1 | TRDMT1 | PYCR2 | KMO | TXNDC12 | MBOAT2 | INPP4A | LPCAT1 | PAFAH2 | SGPL1 | PGP | RPE65 | CA4 |
| PFKFB2 | UGT2A1 | ACSL3 | PDE6C | AK7 | NUDT5 | GPT2 | DNMT3A | ODC1 | WARS2 | GPX1 | GPAM | INPPL1 | PTDSS1 | PLA2G7 | NEU3 | MTHFD1L | RDH8 | CA8 |
| MTMR1 | ALDH9A1 | ACSL4 | PDE6D | ADCY7 | ENTPD1 | DDO | DNMT3B | CNDP1 | OGDHL | GPX2 | LIPG | SYNJ2 | ACHE | PAFAH1B2 | SMPD4 | MTHFD1 | DHRS3 | CA14 |
| PFKFB1 | ALDH3A2 | POLR2G | PDE4D | ADCY5 | PFAS | ACY3 | LDHC | METTL6 | INMT | GPX3 | DGKZ | PIP4K2B | PLA2G3 | EPHX2 | SGMS1 | HAO2 | BCO1 | CA9 |
| AKR1B10 | UGT1A9 | NT5C2 | PDE6A | GMPS | NT5C1B | GAD1 | SMS | ALDH3A1 | TDO2 | IDH2 | DGKE | PLCD3 | ETNK1 | CYP2E1 | ACER3 | HYI | RDH10 | CA1 |
| FPGT | ALDH7A1 | POLR2H | PRIM1 | ADCY10 | APRT | GAD2 | LDHB | BUD23 | HAAO | GPX4 | DGKD | PIKFYVE | PCYT1A | ALOX5 | CERK | ACO2 | CYP26A1 | CA13 |
| KHK | UGT2B11 | ENPP3 | PRIM2 | POLR3K | PRPS1L1 | GOT2 | ENOPH1 | HAL | KYNU | IDH1 | DGKH | IMPA1 | JMJD7-PLA2G4B | CYP2J2 | SMPD2 | SUCLG2P2 | CYP26C1 | SULT1E1 |
| FBP1 | UGT2B10 | POLR2E | AK5 | POLR1B | PPAT | AGXT | CDO1 | HNMT | AADAT | OPLAH | MBOAT1 | IMPA2 | CHPT1 | AKR1C3 | ENPP7 | MCEE | CYP2A13 | SULT1A4 |
| MTMR2 | UGT2B7 | POLR2F | GMPR | PNP | NME7 | GLUL | APIP | DDC | TPH2 | GCLM | GK | PIP5K1A | PLA2G6 | PTGIS | DEGS1 | PCCB | CYP26B1 | SULT1A3 |
| HK2 | UGT2B4 | ENPP1 | NME5 | RRM2B | NT5C | NIT2 | LDHAL6B | ALDH1A3 | ACMSD | ANPEP | DGAT2 | PIP5K1B | PLA2G2E | ALOX12B | SGPP1 | PCCA | DHRS9 | CHST11 |
| HK3 | UGT2A3 | XDH | PDE7B | PDE1A | ALLC | GOT1 | TAT | METTL2B | HIBCH | GSTO2 | GK2 | ISYNA1 | PLA2G10 | CYP4F2 | NEU4 | SUCLG1 | ALDH1A1 | SULT1A1 |
| HK1 | UGT1A4 | POLR2I | PDE5A | POLR2J2 | ZNRD1 | ALDH4A1 | AHCYL1 | ALDH3B1 | MLYCD | G6PD | AGK | INPP5J | LPCAT3 | CYP2C18 | ACER1 | SUCLA2 | RETSAT | SULT2B1 |
| ALDOA | UGT1A1 | POLR2J | POLD4 | HPRT1 | TYMS | ALDH5A1 | MTAP | ALDH3B2 | GGT7 | AMY2B | TKFC | INPP5K | PLA2G2A | PTGES2 | ASAH1 | MMUT | RDH12 | SUOX |
| ALDOC | UGT2B17 | POLE3 | GUCY1A2 | GART | TXNRD1 | ASS1 | MAT2B | HEMK1 | CSAD | UXS1 | AGPAT3 | SYNJ1 | ETNK2 | ALOX15B | SMPD1 | SUCLG2 | LRAT | BPNT1 |
| ALDOB | UGT1A3 | ADSS | FHIT | PAPSS1 | DHODH | ASNS | LDHA | LCMT2 | GGT5 | GYS1 | AWAT2 | PLCD4 | PLA2G4A | ALOX15 | B4GALT6 | ACSS3 | RDH11 | CHST13 |
| MTMR7 | UGT2B15 | PRPS2 | ADCY3 | PAPSS2 | UPRT | ASPA | MTR | UROC1 | GGT1 | GYS2 | AGPAT4 | ITPKA | PLA2G5 | PTGS2 | SPTLC2 | AACS | CYP2A6 | SULT1A2 |
| TSTA3 | CPT1A | ADSL | ADCY2 | NT5M | DTYMK | ALAS1 | MAT1A | TRMT11 | BAAT | GBA3 | PNLIPRP2 | ITPKB | PLA2G12B | PTGS1 | SPTLC1 | OXCT1 | CYP2A7 | CHST12 |
| AKR1B1 | CPT1C | NME6 | ADCY1 | ADCY4 | CTPS1 | ALAS2 | AHCYL2 | FTCD | GGT6 | GUSB | PNLIPRP1 | ITPK1 | PLA2G4B | ALOX12 | SMPD3 | BDH1 | DHRS4 | CYP2F1 |
| SORD | ACADS | POLR1D | POLA1 | IMPDH1 | CAD | GLYCTK | MAT2A | AMDHD1 | ADO | TREH | PNLIP | CDIPT | PTDSS2 | CYP2C9 | KDSR | ACSM1 | ALDH1A2 | AKR1C4 |
| GMPPA | ACADSB | PNPT1 | PDE3A | IMPDH2 | CMPK2 | MAOB | MPST | LCMT1 | SEPHS1 | AMY1A | DGAT1 | INPP4B | PLA2G2F | CYP2C19 | UGT8 | HMGCL | DHRS4L2 | EPHX1 |
| PFKP | ACADL | POLR2K | PDE2A | POLR3H | DCTD | MAOA | LDHAL6A | HDC | SEPHS2 | AMY1B | LPL | PLCE1 | PCYT2 | CYP2C8 | ASAH2 | ACSM3 | RDH16 | CYP2S1 |
| GMPPB | ACADM | PDE11A | PDE1C | POLR2J3 | UCK1 | AOC2 | DNMT3L | HPD | MARS2 | AMY1C | DGKQ | ALDH6A1 | CDS1 | CBR1 | ACSS2 | ACSM4 | RDH5 | AKR1C2 |
| GCK | CYP4A11 | POLD3 | ADSSL1 | POLR1A | UPB1 | GATM | AZIN2 | AOX1 | SCLY | AMY2A | DGKB | PIP5K1C | PGS1 | CYP2B6 | PCK2 | HMGCS2 | PNPLA4 | AKR1C1 |
| GALK1 | ACAT2 | POLR2L | ADA | POLR3B | TXNRD2 | SDS | ARG1 | TYR | MARS | PYGB | DGKG | PIK3C3 | LYPLA1 | CBR3 | ME3 | HMGCS1 | COX10 | DHDH |
| GLB1 | ACADVL | AK1 | ENTPD8 | GUCY1B1 | CMPK1 | AOC3 | ARG2 | HGD | GSTP1 | PYGM | AKR1A1 | PIK3C2B | PLA2G1B | CYP2U1 | GRHPR | BDH2 | ALAD | CYP2D6 |
| GALE | ACAT1 | NME1-NME2 | POLE2 | PKLR | UPP2 | GNMT | CKMT1A | COMT | GSTT2 | PYGL | DGKA | PIK3C2G | CDS2 | LTA4H | PDHB | OXCT2 | CPOX | FMO4 |
| B4GALT1 | ACAA2 | AK2 | PDE8A | GUCY2C | CTPS2 | SHMT1 | P4HA2 | GSTZ1 | GSTT1 | PGM2L1 | PLPP3 | PIP4K2A | PHOSPHO1 | PTGES | PCK1 | ACSM2A | BLVRA | FMO1 |
| PGM2 | HADH | AK4 | PDE9A | ENTPD4 | CDA | GCAT | CKB | DBH | PGD | AGL | LIPC | PIK3C2A | TAZ | CYP4F3 | PDHA1 | ACSM5 | BLVRB | FMO2 |
| LALBA | HADHB | POLA2 | PDE1B | PKM | DUT | PHGDH | NOS3 | TYRP1 | GSTO1 | SI | DGKI | PTEN | GNPAT | LTC4S | PDHA2 | L2HGDH | UROS | FMO3 |
| MGAM | HADHA | CANT1 | NUDT9 | GUCY1A1 | UCKL1 | DMGDH | NOS2 | MIF | GSTA5 | GBE1 | LCLAT1 | PIP4K2C | LPCAT4 | HPGDS | HAGH | ACP4 | HCCS | FMO5 |
| GALT | CYP4A22 | AMPD1 | POLD1 | GUK1 | DPYS | SHMT2 | NOS1 | NAA80 | MGST2 | GPI | GPAT3 | MINPP1 | PLA2G4E | PTGDS | LDHD | ACP6 | FTMT | CES2 |
| G6PC2 | ADH7 | AMPD3 | GDA | GUCY2F | DPYD | SRR | P4HA1 | PNMT | MGST1 | AMDHD2 | MGLL | IPPK | CHKA | TBXAS1 | HAGHL | RFK | MMAB | TPMT |
| GLA | ADH6 | AMPD2 | POLD2 | ADK | TYMP | PSAT1 | AOC1 | DCT | MGST3 | GNPDA2 | GPAT4 | PIK3CA | LYPLA2 | CYP3A5 | ACSS1 | ACPP | FECH | NAT1 |
| GANC | ACSL6 | NME4 | POLE | ITPA | UMPS | BHMT | LAP3 | TPO | GSTA3 | GNPNAT1 | PNPLA3 | PIK3CB | PLA2G2C | CYP3A4 | ACYP2 | FLAD1 | HMOX1 | CES5A |
| LCT | ADH1B | GUCY2D | PDE6B | POLR1C | UCK2 | GLDC | AGMAT | TH | GSTM1 | NANS | PLPP1 | PIK3CD | PLA2G2D | CYP3A43 | ME1 | ACP1 | HMOX2 | CES1 |
| GALK2 | ADH1C | NME1 | PDE7A | URAD | TK1 | DLD | PYCR1 | FAH | GPX7 | CYB5R3 | PLPP2 | PLCG1 | LPCAT2 | CYP3A7 | ACYP1 | ACP2 | PPOX | NAT2 |
| G6PC | ADH4 | ATIC | PRPS1 | POLE4 | TK2 | GAMT | ALDH18A1 | PAH | GSTA4 | NANP | AGPAT1 | PLCG2 | PLA2G12A | FADS2 | ME2 | ACP5 | FTH1 |  |
| UGP2 | ECHS1 | NME2 | POLR1E | RRM1 | UPP1 | PSPH | SAT1 | PRDX6 | GPX6 | CYB5R1 | AGPAT2 | PLCZ1 | PLD1 | GAL3ST1 | MDH2 | NMNAT1 | UROD |  |

Data Source: We downloaded the KEGG signal pathway data by accessing the GSEA database, and extracted the metabolism-related signal pathways (https://www.gsea-msigdb.org/gsea/downloads.jsp), which mainly included three signal pathways:

(1). KEGG_METABOLISM_OF_XENOBIOTICS_BY_CYTOCHROME_P450

(2). KEGG_DRUG_METABOLISM_CYTOCHROME_P450

(3). KEGG_DRUG_METABOLISM_OTHER_ENZYMES.

**Supplementary Table 4.** The final 45 DEmRNAs (or DEGs).

| **Gene** | **entrezID** | **Gene** | **entrezID** |
| --- | --- | --- | --- |
| PFKFB2 | 5208 | ANAPC13 | 25847 |
| MMP2 | 4313 | EMC2 | 9694 |
| ARHGEF37 | 389337 | UBE2Q1 | 55585 |
| LAMC1 | 3915 | MAPRE1 | 22919 |
| KCNAB1 | 7881 | PHF3 | 23469 |
| AQP1 | 358 | PKHD1 | 5314 |
| MYH9 | 4627 | NTRK2 | 4915 |
| 44082 | NA | MAPK1 | 5594 |
| TSPYL4 | 23270 | ATRN | 8455 |
| RAB14 | 51552 | TRADD | 8717 |
| SSX2IP | 117178 | SURF1 | 6834 |
| PLEKHA1 | 59338 | VMA21 | 203547 |
| CNP | 1267 | FOS | 2353 |
| PAPPA | 5069 | C1orf21 | 81563 |
| HRH1 | 3269 | PVALB | 5816 |
| ZFAND2B | 130617 | PCBP1 | 5093 |
| PREX2 | 80243 | SERPINA5 | 5104 |
| MTUS1 | 57509 | SLFN12L | 100506736 |
| B4GALT7 | 11285 | MAFB | 9935 |
| TTL | 150465 | FUT4 | 2526 |
| HIF1A | 3091 |  |  |
| MAP1A | 4130 |  |  |
| SDHAF2 | 54949 |  |  |
| ELK4 | 2005 |  |  |
| SCIMP | 388325 |  |  |

**Supplementary Table 5.** The details for actual gene IDs and GO descriptions.

| **ONTOLOGY** | **ID** | **Description** | **pvalue** | **p.adjust** | **qvalue** | **geneID** |
| --- | --- | --- | --- | --- | --- | --- |
| BP | GO:0007611 | learning or memory | 1.50E-06 | 0.002039 | 0.001592 | KCNAB1/HRH1/HIF1A/MAP1A/NTRK2/MAPK1/FOS |
| BP | GO:0050890 | cognition | 3.93E-06 | 0.002667 | 0.002083 | KCNAB1/HRH1/HIF1A/MAP1A/NTRK2/MAPK1/FOS |
| BP | GO:0007612 | learning | 1.78E-05 | 0.008044 | 0.006282 | HRH1/HIF1A/MAP1A/NTRK2/FOS |
| BP | GO:0008306 | associative learning | 2.80E-05 | 0.009481 | 0.007404 | HRH1/HIF1A/MAP1A/FOS |
| BP | GO:0034614 | cellular response to reactive oxygen species | 3.61E-05 | 0.009795 | 0.007649 | MMP2/AQP1/PLEKHA1/MAPK1/FOS |
| BP | GO:0034599 | cellular response to oxidative stress | 5.48E-05 | 0.011521 | 0.008997 | MMP2/AQP1/PLEKHA1/HIF1A/MAPK1/FOS |
| BP | GO:0006979 | response to oxidative stress | 5.95E-05 | 0.011521 | 0.008997 | MMP2/AQP1/PLEKHA1/HIF1A/MAPK1/ATRN/FOS |
| BP | GO:0007565 | female pregnancy | 6.82E-05 | 0.011559 | 0.009027 | MMP2/PAPPA/UBE2Q1/MAPK1/FOS |
| BP | GO:0044706 | multi-multicellular organism process | 0.000135 | 0.020352 | 0.015893 | MMP2/PAPPA/UBE2Q1/MAPK1/FOS |
| BP | GO:0000302 | response to reactive oxygen species | 0.000166 | 0.021035 | 0.016427 | MMP2/AQP1/PLEKHA1/MAPK1/FOS |
| BP | GO:0060324 | face development | 0.000171 | 0.021035 | 0.016427 | MMP2/PLEKHA1/MAPK1 |
| BP | GO:0014065 | phosphatidylinositol 3-kinase signaling | 0.000336 | 0.037943 | 0.029631 | PLEKHA1/PREX2/NTRK2/MAPK1 |
| CC | GO:0120111 | neuron projection cytoplasm | 3.33E-05 | 0.006732 | 0.005262 | KCNAB1/HIF1A/MAP1A/MAPK1 |
| CC | GO:0001931 | uropod | 0.000357 | 0.02403 | 0.018783 | MYH9/SCIMP |
| CC | GO:0031254 | cell trailing edge | 0.000357 | 0.02403 | 0.018783 | MYH9/SCIMP |
| CC | GO:0031143 | pseudopodium | 0.000619 | 0.031251 | 0.024427 | CNP/MAPK1 |
| CC | GO:0005819 | spindle | 0.000912 | 0.036225 | 0.028316 | MYH9/MTUS1/MAPRE1/PKHD1/MAPK1 |
| CC | GO:0032838 | plasma membrane bounded cell projection cytoplasm | 0.001076 | 0.036225 | 0.028316 | KCNAB1/HIF1A/MAP1A/MAPK1 |
| CC | GO:0072686 | mitotic spindle | 0.001727 | 0.049614 | 0.038782 | MAPRE1/PKHD1/MAPK1 |
| CC | GO:0030863 | cortical cytoskeleton | 0.002063 | 0.049614 | 0.038782 | MYH9/MAPRE1/PVALB |
| CC | GO:0032839 | dendrite cytoplasm | 0.002211 | 0.049614 | 0.038782 | KCNAB1/MAPK1 |

**Supplementary Table 6.** The details for actual gene IDs per pathway (KEGG).

| **ID** | **Description** | **pvalue** | **p.adjust** | **qvalue** | **geneID** |
| --- | --- | --- | --- | --- | --- |
| hsa04010 | MAPK signaling pathway | 0.00079 | 0.041144 | 0.028973 | ELK4/NTRK2/MAPK1/TRADD/FOS |
| hsa05167 | Kaposi sarcoma-associated herpesvirus infection | 0.00129 | 0.041144 | 0.028973 | HIF1A/MAPK1/TRADD/FOS |
| hsa05130 | Pathogenic Escherichia coli infection | 0.001367 | 0.041144 | 0.028973 | MYH9/MAPK1/TRADD/FOS |
| hsa05235 | PD-L1 expression and PD-1 checkpoint pathway in cancer | 0.001506 | 0.041144 | 0.028973 | HIF1A/MAPK1/FOS |
| hsa04657 | IL-17 signaling pathway | 0.001763 | 0.041144 | 0.028973 | MAPK1/TRADD/FOS |
| hsa01522 | Endocrine resistance | 0.001987 | 0.041144 | 0.028973 | MMP2/MAPK1/FOS |
| hsa05231 | Choline metabolism in cancer | 0.001987 | 0.041144 | 0.028973 | HIF1A/MAPK1/FOS |
| hsa04928 | Parathyroid hormone synthesis, secretion and action | 0.002486 | 0.041144 | 0.028973 | MAPK1/FOS/MAFB |
| hsa04659 | Th17 cell differentiation | 0.002554 | 0.041144 | 0.028973 | HIF1A/MAPK1/FOS |
| hsa04668 | TNF signaling pathway | 0.002908 | 0.042166 | 0.029693 | MAPK1/TRADD/FOS |
| hsa04919 | Thyroid hormone signaling pathway | 0.003619 | 0.044279 | 0.03118 | PFKFB2/HIF1A/MAPK1 |
| hsa05020 | Prion diseases | 0.003664 | 0.044279 | 0.03118 | LAMC1/MAPK1 |
| hsa04926 | Relaxin signaling pathway | 0.004334 | 0.047438 | 0.033405 | MMP2/MAPK1/FOS |
| hsa05219 | Bladder cancer | 0.005003 | 0.047438 | 0.033405 | MMP2/MAPK1 |
| hsa04210 | Apoptosis | 0.005025 | 0.047438 | 0.033405 | MAPK1/TRADD/FOS |
| hsa04915 | Estrogen signaling pathway | 0.005235 | 0.047438 | 0.033405 | MMP2/MAPK1/FOS |

**Supplementary Table 7.** The average expression.

| **RNAs** | **Control** | **IDD** | ***p*-value** |
| --- | --- | --- | --- |
| lncR: AC063977 | 6.572±0.371 | 3.012±0.377 | <0.001 |
| miR: miR-338-3p | 0.582±2.475 | 7.317±2.469 | 0.004 |
| mR: PKFKB2 | 7.515±0.413 | 4.315±0.309 | <0.001 |

Abbreviations: IDD, Intervertebral disc degeneration; lncR, long noncoding RNAs; miR, microRNAs; mR, messenger RNA.

^1^**Address:** Department of Orthopedics, The Second Affiliated Hospital of Xi'an Jiaotong University, Xi'an, China.

^✉^**Corresponding author:**

Address correspondence to Dr. Haopeng Li at Department of Orthopedics, The Second Affiliated Hospital of Xi'an Jiaotong University, Xi'an 710004, China.

Tel: +86-29-87679584

E-mail: lihaopeng3993@163.com
